# Single perineal incision placement of artificial urinary sphincter with cadaveric correlation of sub-dartos pump placement

**DOI:** 10.1590/S1677-5538.IBJU.2017.0097

**Published:** 2018

**Authors:** Cooper R. Benson, Hajar I. Ayoub, O. Lenaine Westney

**Affiliations:** 1 University of Texas Health Science Center At Houston McGovern Medical School, USA; 2 University of Texas MD Anderson Cancer Center, USA

**Keywords:** Urinary Incontinence, Stress, Urinary Sphincter, Artificial, Prostatic Neoplasms

## Abstract

**Purpose:**

We present a novel AUS implantation technique using a single perineal incision for single device placement or in combination with an inflatable penile prosthesis (IPP). Urinary and sexual dysfunction following the management of prostate cancer has a significant impact on the quality of life of our patients. While there are marginal changes in the prosthetic devices, we strive to reduce post-operative morbidity while maximizing efficacy.

**Materials and Methods:**

We retrospectively reviewed the outcomes of 6 patients who underwent single perineal incision placement of a virgin AUS in 2014, 3 with simultaneous IPP placement. In all cases, the pressure regulating balloons (PRB) were placed in a high sub-muscular ectopic position and the pumps were placed into a sub-dartos pouch through the perineal incision, which was also validated using a cadaveric model.

**Results:**

The mean patient age was 61 (SD, 7.5 years) with mean body mass index of 31 (SD, 5.9). The average pre-operative pad usage was 7.7 (SD 1.63) pads per day. The mean follow-up was 13.9 months (SD 9.45). Four out of the six patients reported utilizing ≤1 pad daily at follow-up. The one patient who was not initially dry required downsizing of his cuff to 3.5cm; the remaining patient was lost to follow-up. There were no identifiable perioperative or post-operative complications.

**Conclusions:**

We present our initial report of using a single perineal incision for AUS implantation with a validated sub-dartos pump location, which is safe and effective for implantation of an AUS as a single or double implantation in well-selected patients.

## INTRODUCTION

The primary goal in the management of urinary incontinence and erectile dysfunction related to the treatment of prostate cancer is the improvement of long-term quality of life of our patients. Urinary incontinence following radical prostatectomy affects 3%-60% patients and may significantly impact quality of life (1). Sanda et al. found that, at 12 months following prostatectomy, 24% of patients were using pads and 8% found it as a significant problem (2). The artificial urinary sphincter (AUS), first introduced by Scott et al. in 1974, remains a mainstay in the management of post-prostatectomy stress urinary incontinence (SUI) (3). Yet, there has been continued refinement in the technique for implantation in an effort to decrease patient morbidity and discomfort.

Traditionally, the technique for the implantation of the AUS device utilizes two incisions: a perineal and an inguinal incision. This allows for bulbar urethral placement of the cuff and a retropubic location for the pressure regulating balloon (PRB) into the space of Retzius by piercing transversalis fascia (4). In 2003, Wilson et al. introduced a single incision technique for placement of AUS through a transverse scrotal incision (5). Subsequently, Wilson and Delk described ‘ectopic’ placement of the PRB between transversalis fascia and rectus muscles, which would avoid potential problems placing the PRB in the space of Retzius (6). Wherein they describe ectopic placement of the PRB from both transverse scrotal and perineal approaches (6). Ectopic placement avoids the potential hazards of placement into a previously operated field or radiated retropubic space, including injury or obstruction to surrounding vasculature or organs (intestines, bladder, ureter).

Anecdotally, placement of the control pump via a perineal incision can be complicated by pump migration within the scrotum and perineum if a true sub-dartos pouch is not created. The failure to replicate the same placement obtained either with sub-scarpal placement from an inguinal incision or sharp dissection from a scrotal incision can result in instability of the pump location, which may require revision to correct.

Previously, our preferred surgical approach for AUS implantation utilizes two incisions, a perineal incision and a counter lower abdominal incision. We now demonstrate the feasibility of a single perineal incision placement of an AUS.

## MATERIALS AND METHODS

We performed a retrospective review of six patients with stress urinary incontinence undergoing AUS placement through a single perineal incision performed by a single surgeon (OLW) between June 2014 and December 2014 at MD Anderson Cancer Center. Institutional review board approval was obtained for the study and informed consent was obtained from all patients included in this study. All patients underwent routine pre-operative evaluation including 24-hour pad test, urodynamics and office cystoscopy. All patients were virgin AUS placements; however, half (3) underwent simultaneous placement of inflatable penile prosthesis (IPP). Patients who had untreated inguinal hernia or prior inguinal herniorrhaphy were excluded, as well as those patients whose external inguinal rings were inaccessible due to anatomic distance. We utilized the 61-70cm H_2_O PRB filled with 23cc of normal saline in all patients and cuff sizes varied between 3.5-5cm.

**Figure 1:**
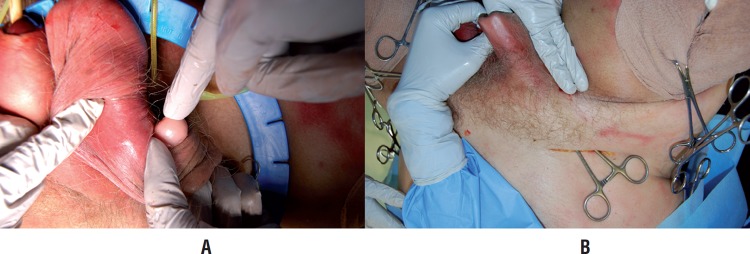
A) Standard pump placement through counter incision B) Pump placement into sub-dartos pouch through perineal incision into the same space.

Additionally, we utilized a cadaveric model to demonstrate the reproducibility of creating the sub-dartos pouch for the AUS pump through the perineal approach, which was identical to the location with traditional placement through the two-incision technique (Figure-1).

## SURGICAL TECHNIQUE

All components of the AMS 800™ AUS (Minnetonka, Minnesota) device were placed through a single perineal incision. After placement of a 12 or 14F foley catheter, a standard midline perineal incision is made. Dissection is performed down to the level of the bulbospongiosus muscle followed by exposure of the corpus spongiosum and bulbar urethra. A Lone Star^®^ retractor with blunt hooks exposes the surgical field. A combination of sharp and blunt dissection is used to mobilize approximately a 2cm segment of the proximal bulbar urethra circumferentially with subsequent measurement of the urethra with a cuff sizer.

**Figure 2:**
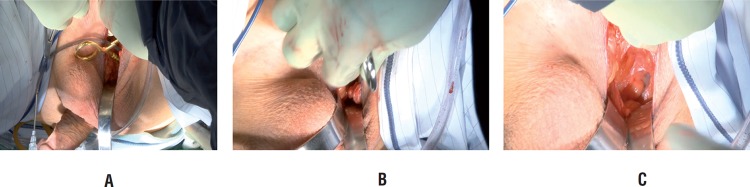
A) Retractor placed into external inguinal ring through perineal incision B) Placement of ring clamp into external inguinal ring C) Filling of positioned PRB.

After the urethra is sufficiently mobilized, our attention turns to palpating the external inguinal ring through the perineal incision. Once a finger is placed within the external inguinal ring, a pediatric deaver retractor retracts the anterior wall of the canal, and a ring forceps is advanced cephalad and medial to the spermatic cord spreading to create a potential space between transversalis fascia and rectus muscle (Figures 2a and b). Once this space has been created, a coated ring forceps is utilized to advance the PRB into the prepared ectopic space followed by inflation with 23cc of injectable saline (Figure-2c). The tubing is occluded with hemostat. Interrupted 3-0vicryl sutures are placed around the entrance of the PRB into the ectopic space to prevent migration.

**Figure 3:**
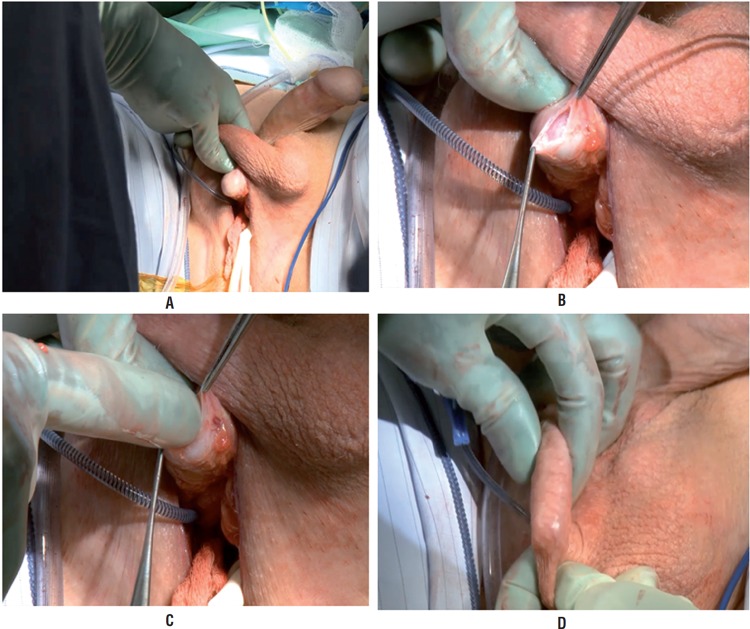
A) and B) Dissection of internal spermatic fascia with Metzenbaum scissors C) Finger entry into sub-dartos pouch D) Pump location in newly created dartos pouch.

Attention is turned to creating a sub-dartos pouch for the AUS pump. Blunt dissection is utilized to deviate the tunical sac medially. The right hemi-scrotal skin is inverted through the perineal incision (Figure-3a). The internal spermatic fascia is incised until the dartos fibers are visualized (Figure-3b). A finger is placed within this incision and used to bluntly create a space for the pump by reverting the scrotal skin (Figure-3c). The skin is inverted through the incision and the pump is placed within this space with the pump positioned within the pouch. Per standard, the deactivation button is positioned laterally (Figure-3d). The position is stabilized with a Babcock clamp.

The cuff is then placed around the urethra using a right angle clamp and clipped into position. The tubing from the cuff is then passed with a needle passer through the bulbospongiosus muscle and through the Colle's fascia into the same plane as the pump tubing. The tubing length is planned to assure that the connections will reside in an inguinal location. The Quick-Connect system is used to seal the connections. The tubing is tucked superiorly to an inguinal location. A suture is placed to assure that the tubing does not prolapse into the perineum.

The bulbospongiosus is then closed in a running fashion, followed by Colle's fascia and then the perineal skin. The Babcock is then removed and the device is cycled and subsequently deactivated.

## RESULTS

A total of 6 AUS devices were placed using the single perineal incision technique. The etiology of the urinary incontinence was prostate cancer treatment related in all cases. All six patients underwent radical prostatectomy (4 robotic assisted and 2 radical retropubic), of which 5 were treated with radiation either as their initial treatment or in the post-operative salvage setting.

The average patient age was 61 (SD 7.5 years) and average BMI 31 (SD 5.9). The average pre-operative pad usage was 7.7 pads daily (SD 1.63), 24-hour pad weight was available for 4 of the patients with an associated mean 24 hour pad weight of 517.6g (SD, 605g). Four patients underwent urethral procedures prior to AUS implantation: 3 patients underwent direct visualized internal urethrotomy for bladder neck contracture and one patient required buccal mucosal graft urethroplasty for urethral diverticulum. Pre-operative cystoscopy to document the stability of urethra prior to AUS implantation was performed in all cases. The average operative time was 101 minutes (SD, 27 minutes) for the entire cohort including cases with simultaneous implantation of AUS and IPP; however, the 3 patients that had AUS alone, the mean operative time was 81 minutes (SD, 17 minutes).

The mean follow-up for the cohort was 13.9 months (SD 9.45). Four patients met criteria for “socially dry” (1 pad or less per day), of which two were wearing zero pads at last follow-up. There was one patient who continued to have significant incontinence, wearing 10 pads daily after initial AUS placement. Of note, his pre-operative 24-hour pad weight was 1700grams. Initially, he had a 5cm cuff placed and underwent downsizing of his cuff to 3.5cm and was recently activated. One patient was lost to follow-up after activation of his device. There were no reported perioperative complications by any patient over the follow-up period, including infections, erosions, PRB herniation or pump migration. Further no device related morbidity occurred in patients with simultaneous IPP placement, and all had satisfactorily functioning IPP and AUS devices.

## DISCUSSION

The AUS continues to be relevant and the standard management for moderate-to-severe post-prostatectomy stress urinary incontinence (3). Despite few modifications in the AMS 800 Urinary Control System itself over the last decades, there has been advancement in our understanding of the function of the device as well as novel implantation considerations, with the goal of minimizing complications.

Wilson and Delk initially described utilizing a single transverse scrotal incision to implant the AUS, with ventral retraction rather than division of the bulbocavernosus muscle. They found that 66% of patients were completely dry with mean follow-up of 12 months, and compared this to the traditional two-incision approach and found similar continence rates (5). The trans-scrotal approach was initially utilized for revision and reimplantation cases to avoid a scarred perineum; however, it was adopted as a more efficient approach in the primary setting. Despite this, others have been critical of the outcomes with this technique.

Henry et al. suggested that the penoscrotal placement of the AUS had inferior functional outcomes compared to the originally described perineal approach, which they evaluated in both primary and revision settings (7, 8). In a retrospective series of virgin implantations, they found that that 7 of 25 patients (28%) with scrotal incision compared to 17 of 30 patients (56.7%) with a perineal incision were completely dry without pad usage (p=0.03) (7). However, in regards to social continence, defined as wearing one pad or less daily, there was not a significant difference between the groups (7). Overall, including both initial placements and revisions, there was a significant difference in completely dry rate between the perineal and scrotal approaches (p=0.01) (7). In a multicenter study including 158 patients, the perineal incision group was more likely to be completely dry than the scrotal incision group (44.1% versus 27.4%, p=0.04) (8). The scrotal incision group was also more likely to require tandem cuff placement for continued incontinence after initial implantation (10% versus 1.4%, p=0.04) (8). There was no difference in AUS device durability nor rates of complications, between the two techniques (8). Similarly, our preferred approach for virgin AUS placement is perineal to facilitate access to the proximal bulbar urethra.

Wilson and Delk initially described the ectopic placement of the PRB to avoid the risks associated with blind puncture of the transversalis fascia and placement into the retropubic space (6). The ectopic location is a potential space developed between transversalis fascia and posterior rectus muscle using blunt finger dissection through the external inguinal ring. They described ectopic placement both via perineal and transverse scrotal incisions without need for a second counter incision (6). Morey et al., also described ectopic submuscular placement using a Foerster clamp to develop the potential space (9). However, in their series, a scrotal counter incision was used for ectopic placement of the PRB, whereas we utilize a single perineal incision. Further, Singla et al., compared the outcomes of the AUS with an ectopic PRB versus a PRB within the retropubic space (10). There were no significant differences in continence outcomes (88% versus 81% p=0.11), erosion rates (8% versus 9% p=0.66) and need for revisions (8% versus 13% p=0.16) in this series and similar rates of explantation (10).

There is a paucity of high quality evidence regarding AUS implantation; there are non-uniform outcome measures (both objective and subjective) and definitions of continence, making it difficult to interpret the evidence as a whole (11-14). A systematic review by Van der Aa et al., 79% patients included were socially continent and 43.5% completely dry without any pads and a 26% reintervention rate (11). Overall, the early results of our technique are generally consistent with expected outcomes. Our primary concerns were related to the comparability of the pump placement to standard techniques. By the time of activation, the sub-dartos location of the pump should be stabilized, especially with more consistent manipulation by the patient. Thus, the likelihood that dislocation problem would develop decreases with time. Assuming that the continence and long-term complications are similar to other techniques, there are several benefits of a single perineal incision. There is expected improvement in patient discomfort and bother with a single incision, while saving the surgeon the need to open and close a second site and thus shortening the time of the procedure.

The limitations of this study include the small sample size, the retrospective nature and limited follow-up regarding single perineal incision AUS implantation. Despite this, we believe this technique is reproducible, safe, and effective in an appropriately selected patient. This study is also limited by the lack of patient satisfaction outcomes and comparison with standard implantation techniques. The endpoint of interest is stability and functionality of the pump due to the mechanism of sub-Dartos pouch formation considering that the cuff and reservoir placement are consistent with our standard technique. Thus, the follow-up is sufficient to determine whether any substantial problems are encountered on the basis of this variation in technique. We will continue to monitor these patients to confirm that the long-term outcomes (e.g. continence, pump migration, PRB migration) are consistent with their two incision counterparts.

## CONCLUSIONS

This study demonstrates the feasibility of a single perineal incision for AUS placement in the properly selected patient. Utilization of a single perineal incision for AUS placement is safe and effective. Longer follow-up will be necessary to confirm no pump related mechanical problems specifically related to this technique.
